# Smartphone Instrumented 30-Second Chair Stand Recovers Body Composition and Strength Biomarkers

**DOI:** 10.21203/rs.3.rs-9931907/v1

**Published:** 2026-07-02

**Authors:** Colin Barry, Edward Jay Wang, David Wing

**Affiliations:** 1 Electrical and Computer Engineering Department University of California San Diego, La Jolla, CA, USA; 2 Exercise and Physical Activity Resource Center, University of California San Diego, La Jolla, CA, USA; 3 School of Public Health, University of California San Diego, La Jolla, CA, USA

## Abstract

The 30-second chair stand test is widely used in geriatric assessment, yet clinical practice only records the repetition count, discarding the movement information within each cycle. This manuscript reveals that a smartphone held at shoulder level during the test can not only count stand–sit cycles, but also recover information related to body composition and strength. In a study of 78 adults, movement features extracted from the smartphone inertial measurement unit (IMU) signal during a chair stand test were used in simple regression models to predict clinical metrics from a dynamometer and dual-energy X-ray absorptiometry (DXA) scanner. Combined with height and weight to compute force and inverse kinematics, the smartphone-derived features predict dominant grip strength with Pearson *R* = 0.83, percent body fat with *R* = 0.62, and total lean mass with *R* = 0.92. Even without height and weight, the IMU-only features significantly exceed the repetition count alone performance for all three outcomes (*p* ≤ 0.02).

## 1 Introduction

The 30-second chair stand test (30s CST) is one of the most widely deployed tools in geriatric and sarcopenia assessment [1]. A patient grips the arms of a chair, stands and sits repeatedly for 30 seconds, and a clinician counts the repetitions. That count, a single integer, is the sole outcome. Other information including fatigue over the 30 seconds, smoothness of movement, and other potential metrics are rarely considered.

This is a striking information loss. Each stand-to-sit cycle is a coordinated neuromuscular event requiring eccentric and concentric loading of the quadriceps, gluteal muscles, and postural stabilizers. The velocity, smoothness, symmetry, and progressive fatigue visible across those 30 seconds reflect the very muscle quality and functional reserve that sarcopenia erodes [2]. Yet standard practice collapses this rich temporal signal into a scalar, discarding everything about *how* the person moved.

Sarcopenia, the age-related loss of skeletal muscle mass, strength, and quality, affects 5–13% of adults aged 60–70 and up to 50% of those over 80 [3], and carries well-documented risk of falls, fractures, disability, and mortality [4]. Traditional assessment relies on dual-energy X-ray absorptiometry (DXA) for muscle mass quantification [5], a modality that is accurate but expensive, equipment-dependent, and confined to clinical or research settings, creating real barriers to early detection and longitudinal monitoring.

Recent clinical guidelines to diagnose sarcopenia in research and care are increasingly utilizing functional assessments like the 30s CST as an essential component [6–8]. These functional assessments have proven correlations with clinical outcomes and allow insight into practical daily movements with little equipment[1], [4].

Smartphones offer an opportunity to expand both the accessibility and the utility of these functional assessments. All modern smartphones carry high-fidelity inertial measurement units (IMUs), accelerometers and gyroscopes, capable of capturing the full kinematic trajectory of every stand-to-sit cycle at sampling rates sufficient to resolve movement dynamics. A participant simply holds their phone during the test they are already performing. No additional equipment, no clinical infrastructure, no change to workflow.

**Figure 1:**
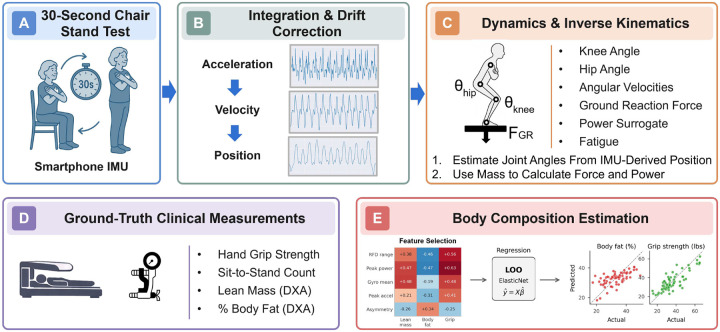
Overview of the study pipeline. Participants performed the 30-second chair stand test while carrying a smartphone at the shoulder level, and phone IMU signals were processed to derive cycle-level dynamics and inverse-kinematics-based joint-angle features. These features were then analyzed for association with clinical metrics (total lean mass, hand grip strength, and percent body fat) and used in cross-validated predictive modeling.

Prior laboratory studies using force-plates, motion-capture systems, or body worn sensors identified some chair-stand kinematic features informative for sarcopenia-relevant physiology. These features fall into three main groups. Force and power: ground reaction force and rate of force development at seat-off [9], and mechanical/leg-extension power during the rise phase [10]. Angular kinematics: peak knee extension angular velocity and trunk elevation rate [11]. Temporal coordination: trunk flexion angle at seat-off and timing of post-stand stabilisation [12]. The feature engineering in this paper targets these three clusters directly from a single shoulder-mounted smartphone IMU, without the force plates or motion capture used in the prior work. As shown in Figure 1, this paper presents a study that compares these smartphone-derived features to clinical measurements of strength and body composition.

The smartphone application is the first highly scalable means to administer the 30s CST, collect data, and generate additional biomarkers. However, the main contribution of this paper is to demonstrate that movement information discarded by standard 30s CST scoring can be recovered from a held smartphone and translated into biomarkers with substantially stronger relationships to strength and body composition than the repetition count alone. These biomarkers can be collected as a passive, zero-burden addition to the 30s CST already administered in millions of clinical encounters annually. By leveraging commodity devices to convert the single-integer outcome into several biomarkers, this approach offers a path from periodic, equipment-burdened snapshots of body composition towards comprehensive muscular health monitoring beyond the clinic.

## 2 Results

### 2.1 Model Performance Across Conditions

**Figure 2:**
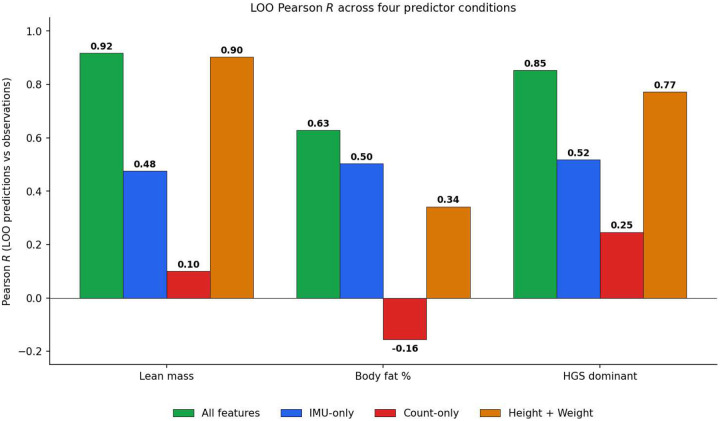
Leave-one-out cross-validated *R*^2^ for each outcome across four predictor conditions: IMU-only (movement features derived solely from the smartphone), all features (IMU plus height, weight, and BMI; BMI is a function of height and weight), count-only (the conventional 30-second chair stand repetition count), and height-and-weight (height and weight only; BMI is included in the all-features model as a derived predictor since it is a function of height and weight). Data labels show *R*^2^ values for each bar.

Figure 2 compares LOO *R*^2^ across all four predictor conditions for each outcome. Full numeric results with 95% bootstrap confidence intervals and Steiger Z-test statistics are provided in Tables 1 and 2.

Across the four conditions, the all-features model (curated movement features plus height, weight, and BMI) achieved the highest predictive performance for every outcome (lean mass Pearson *R* = 0.92, percent body fat *R* = 0.63, dominant HGS *R* = 0.85). The relative contribution of body size versus movement varies sharply by outcome. For lean mass and grip strength the height-and-weight baseline already reaches *R* = 0.90 and 0.77 respectively, so adding the IMU and BMI features on top of body size contributes little for lean mass (*R* = 0.92 vs 0.90) and a modest amount for grip strength (*R* = 0.85 vs 0.77). For body fat the all-features model exceeds the height-and-weight baseline by a meaningful margin (*R* = 0.63 vs 0.34), reflecting that movement and BMI add information about adiposity on top of body size. The scientifically central comparison is the demographic-free IMU-only condition (curated set of demographic-free IMU features (33 features for percent body fat; 37 features including angular velocity for lean mass and grip strength), fit with ridge regression), which measures what the movement signal contributes once body size is removed. In that condition, percent body fat (*R* = 0.50, 95% CI: 0.33–0.65), dominant grip strength (*R* = 0.52), and lean mass (*R* = 0.48) all retained a clear movement-specific signal. Percent body fat shows the most striking contrast: the height-and-weight baseline reaches only *R* = 0.34, the demographic-free movement signal alone reaches *R* = 0.50, and the full all-features model reaches *R* = 0.63, so the predictable body-fat information in the chair stand is carried primarily by movement (with BMI complementing it), not by body size. The count-only baseline was the weakest condition for every outcome (|*R*| ≤ 0.25), confirming that the single repetition count discards most of the recoverable information in the test.

### 2.2 LOO Predicted vs. Actual

**Figure 3:**
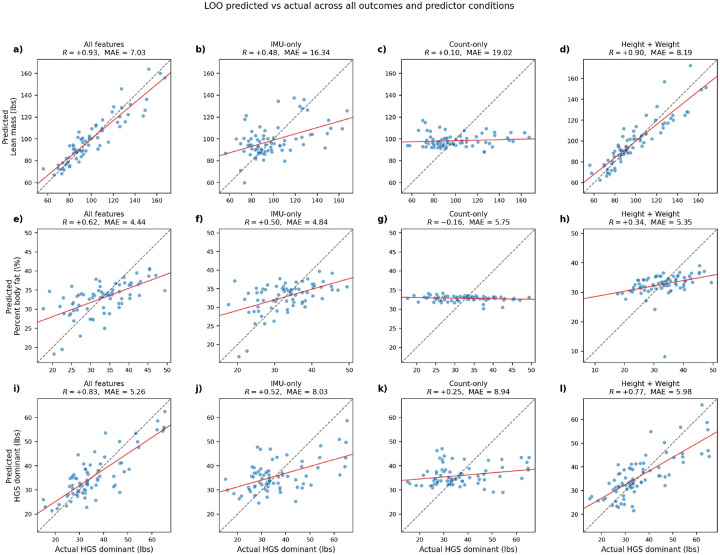
Leave-one-out (LOO) predicted vs. actual values for all three outcomes across the four predictor conditions. Rows correspond to outcomes (top: lean mass; middle: percent body fat; bottom: dominant hand grip strength); columns correspond to predictor conditions (All features; IMU-only; Count-only; Height + Weight). Each subplot shows the Pearson correlation *R* and mean absolute error (MAE) for the LOO predictions, the identity line (dashed), and a linear fit (solid).

Figure 3 shows LOO predicted versus actual values for all three outcomes (lean mass, percent body fat, dominant hand grip strength) across all four predictor conditions. HGS is the functional strength outcome most directly linked to sarcopenia diagnosis under EWG-SOP2 and AWGS criteria; percent body fat illustrates the body composition outcome where movement-specific signal is most interpretable after controlling for body size.

### 2.3 Agreement Analysis

Bland–Altman analysis for the IMU-only condition showed near-zero mean biases for every outcome (lean mass: −0.44 lbs; percent body fat: −0.12%; dominant HGS: −0.34 lbs), indicating no systematic over- or under-prediction. The 95% limits of agreement are wide, however, reflecting the modest *R*2 at *N* = 78: ±45 lbs for lean mass, ±12% for percent body fat, and ±20 lbs for dominant grip strength. These limits are consistent with the MAE values reported in Table 1, the precision at this sample size is suitable for cohort-level effects but too coarse for individual-level prediction; this is consistent with the *R* ≈ 0.5 values reported above. Combined scatter panels for all three outcomes across all four predictor conditions are shown in Figure 3.

### 2.4 Statistical Comparison of Conditions

Steiger’s Z-tests (Table 2) confirmed that the demographic-free IMU-only condition produced significantly higher correlations than the count-only baseline for ALL three outcomes (Steiger Z-test): percent body fat (*p* < 0.001), dominant grip strength (*p* = 0.019), and lean mass (*p* = 0.007). Comparison against the height-and-weight baseline gives a different picture: for lean mass and dominant grip strength, height and weight produce significantly higher correlations with the outcome than the IMU-only movement model (*p* < 0.01), reflecting that body size is the stronger predictor of these body-size and strength measures. For percent body fat the IMU-only model achieves a numerically higher correlation (*R* = 0.50) than the height-and-weight baseline (*R* = 0.34); the Steiger test on dependent correlations is not significant at *p* = 0.27 at *N* = 78, but the difference is in the direction of stronger movement-specific signal for body fat. The 95% bootstrap confidence intervals on the IMU-only *R* (Table 1) are wide at *N* = 78, so these point estimates should be read as preliminary, and replication in a larger cohort is needed to narrow these intervals.

### 2.5 Partial Correlations: Movement-Specific Signal

The partial correlations between IMU features and each outcome after regressing out height, weight, and BMI, isolating movement-specific information content independent of body size. For lean mass, 15 of the 33 curated IMU features retained a statistically significant partial correlation with the outcome after residualising on height, weight, and BMI (*p* < 0.05, two-sided Pearson). The corresponding counts are 12 for percent body fat and 19 for dominant grip strength. The reduction in correlation magnitude from raw to partial *r* was largest for the Power/Impulse features, consistent with those features partially tracking body-size-composition covariance in addition to genuine movement-specific physiology.

### 2.6 Minimal Sufficient Feature Sets

**Figure 4:**
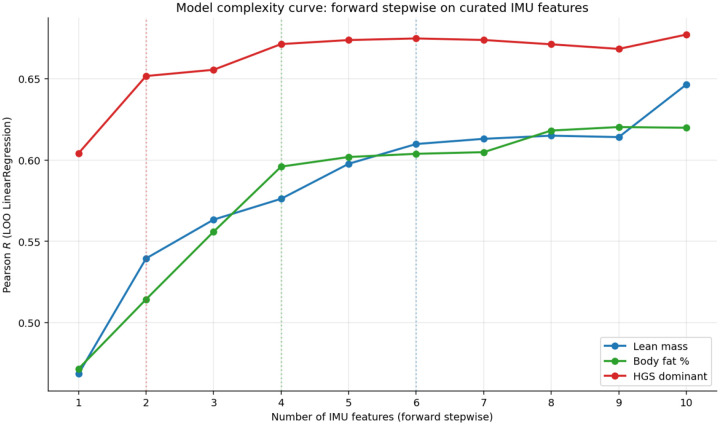
LOO *R*^2^ as a function of the number of IMU features selected by forward stepwise selection. Dotted vertical lines mark the elbow point for each outcome (first *k* where marginal gain < 0.01). Elbow annotations show both the feature count and the movement category abbreviation of the elbow feature (Sp = Speed, Po = Power/Impulse, Sm = Smoothness, Fa = Fatigue, Ki = Kinematics, Cy = Cycle/Consistency).

Figure 4 shows LOO *R*^2^ as a function of model size from forward stepwise selection (*k* = 1 to 10 IMU features). For lean mass, the elbow occurred at *k* = 2 features (*R* = 0.54); for percent body fat at *k* = 4 (*R* = 0.60); and for dominant grip strength at *k* = 1 (*R* = 0.60). Full step-by-step tables listing the feature added at each step with its movement category, description, and marginal *R*^2^ gain are provided in Tables 3–5.

## 3 Discussion

By capturing only a single integer count, standard 30-second chair stand scoring discards a wealth of information about how a person moves. This study asked how much of that discarded information is genuinely about movement rather than body size, by stripping body mass out of the force, power, and impulse features and fixing a single population-average height in the inverse-kinematics features, then using both the linear-acceleration and angular-velocity channels of the IMU (angular features included for lean mass and grip strength, where they carry complementary movement signal, and omitted for percent body fat by a-priori physiological reasoning). Under leave-one-out cross-validation, this demographic-free movement signal predicted dominant grip strength with Pearson *R* = 0.52, percent body fat with *R* = 0.50, and total lean mass with *R* = 0.48, significantly exceeding the count-only baseline for all three outcomes (Steiger *p* ≤ 0.02).

Prediction of total lean mass from movement alone remained well below body size: lean mass is essentially a body-size quantity, although including the angular-velocity IMU channels alongside the linear-acceleration channels lifted IMU-only lean mass from *R* = 0.30 (linear-acceleration only) to *R* = 0.48, the height-and-weight baseline still reaches *R* = 0.90. The all-features model (curated movement features plus height, weight, and BMI) predicts lean mass with *R* = 0.92, barely above the height-and-weight baseline (*R* = 0.90), so the predictive power for lean mass comes almost entirely from body size, not from movement quality. Percent body fat shows the opposite pattern: the height-and-weight baseline reaches only *R* = 0.34, the demographic-free movement signal alone reaches *R* = 0.50, and adding BMI raises the all-features model to *R* = 0.63, indicating that for body fat the chair-stand movement signal carries information about adiposity that body size alone does not provide. Grip strength sits between these extremes, height and weight reach *R* = 0.77, the demographic-free movement model adds another *R* = 0.52 on its own (significantly above count, *p* = 0.019), and the full all-features model reaches *R* = 0.85, indicating that both body size and movement carry meaningful, complementary information about strength. Body fat and grip strength are therefore the outcomes for which the chair-stand movement signal carries genuine, body-size-independent information, and they are the most promising targets for a smartphone-based functional biomarker.

Several limitations should be acknowledged with the specificity their implications warrant.

### Participant demographics.

The participants in this study were largely younger than individuals most susceptible to sarcopenia and, as such, the majority were above commonly used clinical thresholds. The 95% bootstrap CIs on IMU-only *R*^2^ (Table 1) are wide, spanning roughly 0.33 to 1.21 *R*^2^ points at *N* = 78, indicating that point estimates should be interpreted with appropriate caution. Validation in a cohort spanning the sarcopenic range is essential before clinical utility can be claimed.

### DXA measurement variability.

Total lean mass and percent body fat were measured using two different devices, the Hologic Discovery W and the GE Lunar Prodigy. Prior research has demonstrated measurement variation between these systems. For the purposes of this feasibility study the two devices are treated as equivalent, but future investigations moving beyond feasibility toward clinical validation should account for this potential source of measurement error.

### Kinematic model validity.

The two-link planar IK model has not been validated against motion capture or multi-sensor ground truth. Joint angle estimates should be treated as movement-quality indices rather than anatomically precise angles. Sensitivity analyses perturbing segment length assumptions by ±10% and phone placement height by ±10 cm demonstrated that IK-derived features showed limited sensitivity to these variations; non-kinematic IMU features (speed, power, smoothness, fatigue) are less sensitive to exact placement and may be more robust for unsupervised deployment.

### Automated stand–sit count detection.

Across the 78 trials, the smartphone-derived stand–sit count (a prominence-based peak detector on the gravity-aligned vertical acceleration magnitude) reproduced the human-recorded 30-second count with a mean absolute error of 2.2 cycles and a Pearson correlation of *R* = 0.76. Roughly half of the trials (48%) were off by exactly −1 or −2, a signature consistent with the first and/or last stand–sit cycle of the 30-second window not being detected (the boundary cycles begin or end partway through the test, so their acceleration peaks fall outside the prominence-based detector’s effective window). A smaller, larger-magnitude error mode is concentrated in the fastest standers: trials with a stand–sit cycle rate at or above ~ 0.8 cycles/s (n = 13) showed a count MAE of 4.5 cycles versus 1.8 cycles for slower trials, with the adjacent stand and sit peaks merging when they fall closer together than the fixed prominence window can separate. Such fast standers are not the demographic of interest, as individuals who can complete a stand–sit cycle in under a second are unlikely to be near the sarcopenia or frailty thresholds the test is designed to flag. All analyses in this paper used the human-recorded count for the Count-only baseline, so this detection limitation does not affect the reported comparisons; a deployable on-device count would still benefit from an end-of-window correction for boundary cycles and an adaptive prominence/minimum-distance scheme for the small subset of very fast performers.

### Height and Weight

Some of the most significant features involve estimating force or power by multiplying body mass times the IMU derived acceleration. This is unsurprising as body mass and height alone are well correlated with the outcomes, especially lean mass (height-and-weight *R* = 0.90 vs IMU-only *R* = 0.48) because lean mass is a proportion of total body mass. Even so, IMU-only features alone significantly exceed the count-only baseline for every outcome (Steiger *p* ≤ 0.02). Also, the IMUonly features outperform height and weight for predicting percent body fat (height-and-weight *R* = 0.34 vs IMUonly *R* = 0.50), demonstrating the movement information is not dependent on demographic information.

### Single-visit cross-sectional design.

Longitudinal validity, whether these features track changes in body composition over time, is unknown and is a necessary next step for clinical translation.

These caveats notwithstanding, the core result stands: the information routinely discarded by standard 30s CST scoring is recoverable from a held smartphone and contains clinically meaningful signal. The infrastructure required is already in patients’ hands.

## 4 Methods

### 4.1 Data Collection

All data were collected at the Exercise and Physical Activity Resource Center (EPARC) at the University of California, San Diego (UCSD) under IRB approval (#811902). Eighty-five participants were recruited from individuals scheduled for clinical DXA scans at EPARC. Participation was limited to a single visit. Eligibility required English literacy, age 18 or older, and willingness to complete grip-strength and DXA assessments; individuals with neuromuscular disorders (e.g., Parkinson’s disease, multiple sclerosis), conditions impairing hand function (e.g., severe arthritis, carpal tunnel syndrome, peripheral neuropathy), recent upper-extremity injuries, or medications known to affect grip strength were excluded. All participants provided written informed consent before participating in any study activities.

DXA scans were performed on a Hologic Discovery W or GE Lunar Prodigy system, providing reference measurements of total lean mass and percent body fat. Dominant and non-dominant handgrip strength were measured using a Jamar Plus+ digital hand dynamometer (Performance Health, Cedarburg, WI, USA). Demographic and anthropometric data including gender, height, and weight were also recorded. After excluding trials with missing IMU data and those failing internal consistency checks (weight less than measured lean mass; percent body fat deviating by more than 10 percentage points from the lean-mass-derived estimate), the analytic sample comprised *N* = 78 participants. Table 6 summarizes the characteristics of the final analytic sample. The cohort was predominantly female (73.1%) with a mean age of 51.5 ± 16.4 years and a mean BMI of 24.1 ± 4.3 kg/m^2^. Mean 30-second chair stand count was 16.9 ± 5.5 repetitions (range 9–30).

### 4.2 Chair Stand Protocol

Participants performed the 30s CST while holding a smartphone against their chest with arms crossed, positioning the device near shoulder level. A custom application running on a Google Pixel 8 recorded triaxial acceleration and angular velocity throughout the task. Participants were instructed and verbally encouraged to stand fully and sit down as many times as possible within 30 seconds, consistent with standard clinical protocol [1].

### 4.3 Signal Processing and Cycle Detection

#### Preprocessing.

Raw triaxial acceleration channels were linearly interpolated to fill missing samples where sufficient valid data existed; otherwise, invalid values were set to zero. Each axis was zero-centered by subtracting its sample mean to remove DC offset. The sensor frame was reoriented by estimating the gravity vector from the median acceleration over the first 1.0 s of each trial and computing a rotation matrix to align the vertical axis with a consistent world frame. A fourth-order Butterworth low-pass filter with a 2 Hz cutoff was applied to the reoriented signals to remove high-frequency noise while preserving the dynamics of sit-to-stand transitions.

#### Velocity and position estimation.

Mean-subtracted acceleration was numerically integrated to obtain velocity. A 0.1 Hz low-pass filter was applied to the velocity signal to estimate and subtract integration drift before a second integration to obtain position. The resultant (magnitude) acceleration, velocity, and position signals were computed from the three-axis signals and used for cycle detection and trial-level feature extraction.

#### Cycle detection.

Stand cycles were identified as peaks in the resultant position signal with a minimum prominence of 0.2. Rules were applied to retain the first and last peaks at trial boundaries where appropriate. The number of detected peaks corresponds closely to the conventional 30s CST count. For per-cycle analysis, a window spanning 0.4 s before to 1.2 s after each detected peak was extracted.

### 4.4 Feature Engineering

Features were extracted at both the trial level and the cycle level (aggregated per trial). The six feature categories were motivated in part by prior work with sensor-based sit-to-stand: power and impulse features[9, 10, 13, 14]; angular velocity and kinematic features[11, 15]; and timing and consistency features[12, 16]. The present work approximates all three feature clusters from a single held smartphone rather than specialized force-plate or multi-IMU systems. All features are organized into six interpretable categories.

**Speed features** characterize how quickly participants complete each stand and sit phase. Trial-level speed features include maximum resultant velocity, standard deviation and range of resultant velocity, 5th percentile of resultant velocity, and time to complete the first five stands. Per-cycle speed features include mean rise time (trough-to-peak velocity duration) and mean sit time (peak-to-trough duration), computed for each cycle and averaged across the trial. Inverse-kinematics–derived speed features include peak and mean knee and hip angular velocities.

**Power and impulse features** capture the total force effort per repetition. Trial-level features include the standard deviation and range of resultant acceleration and the 85th percentile of resultant acceleration (a proxy for peak force production) Per-cycle peak force surrogates (computed as body mass times peak resultant acceleration) and their rate-of-development counterparts are also computed and recorded; for modelling, the body-mass-normalised siblings (mathematically equivalent to peak acceleration) are the form offered to every condition, with body mass available as a separate predictor so the all-features ElasticNet can recover the multiplicative *F* = *m* · *a* structure through its anthropometric channel rather than through a co-linear weight-scaled feature.. Per-cycle features include mean mechanical impulse (area under the absolute acceleration curve per cycle, scaled by body mass in kg) and mean mechanical power (mean absolute product of acceleration and velocity per cycle).

**Smoothness features** quantify movement coordination. Mean peak jerk (mean peak rate-of-change of acceleration per cycle, in m/s^3^) was computed as a marker of abrupt, poorly coordinated transitions. Trial-level smoothness features include the number of velocity zero-crossings and the proportion of samples with resultant velocity below 0.2 m/s (movement consistency).

**Fatigue features** capture within-test decline across the 30 seconds. Five features were extracted from the inter-peak interval sequence and the per-cycle velocity sequence: fatigue delta (last minus first inter-peak interval), fatigue slope (linear slope of inter-peak intervals over cycles), fatigue ratio (mean interval in the final third of cycles divided by the first third), velocity trend (linear slope of per-cycle peak velocity), and velocity ratio (mean peak velocity in the final third divided by the first third).

**Kinematic features** are derived from a planar two-link inverse kinematics model. The knee was treated as a fixed origin; the thigh (length = 0.345× height) connected the knee to the hip; and the torso (length = 0.50× height) connected the hip to the shoulder (phone location). Shoulder position was obtained by double integration of vertical and anterior–posterior acceleration with drift correction. For each time point, knee and hip joint angles were computed from the shoulder position using the law of cosines. Trial-level kinematic features included mean, standard deviation, minimum, maximum, and range of both knee and hip flexion angles.

**Cycle consistency features** summarize repetition-level regularity. Mean stand asymmetry was defined as the mean normalised absolute difference between rise time and sit time per cycle (0 = symmetric, 1 = entirely asymmetric). The total number of detected stand cycles was also retained as a feature, providing a direct counterpart to the conventional count-based score.

### 4.5 Statistical Analysis

#### Regression targets.

Three outcomes were modeled: total lean mass (lbs), percent body fat, and dominant handgrip strength (HGS). Lean body mass index (LBMI) and non-dominant grip strength were initially considered but produced results essentially identical to lean mass and dominant grip strength respectively (Pearson *r* > 0.95 between paired predictions), so the manuscript reports only the non-redundant outcomes.

#### Predictive model conditions.

Predictive performance was evaluated under four feature-set conditions, designed to isolate the contribution of IMU-derived movement information from body size:

1. *IMU-only (demographic-free)*: movement features derived from the smartphone IMU signal (both linear-acceleration and angular-velocity channels) with all demographic information removed. The angular-velocity features are included for lean mass and grip strength where they carry complementary movement signal, and omitted for percent body fat where they do not. This distinction is essential. Several mechanical features are, by construction, the product of body mass and acceleration (the force surrogates, mechanical power, and impulse all scale as *ma*), and the inverse-kinematics joint angles depend on each participant height through the assumed limb-segment lengths. To prevent body size from entering the movement model implicitly, body mass was divided out of the force, power, and impulse features, and a single population-average height (1.75 m) replaced each participant measured height in the inverse-kinematics computation. The IMU-only condition uses a curated, interpretable set of demographic-free features spanning all six movement categories, fit with ridge regression (RidgeCV) because the features are numerous and correlated.
2. *All features*: the same curated demographic-free movement features used by the IMU-only condition (33 interpretable features spanning the six movement categories), combined with the anthropometric predictors height, weight, and BMI (= 703·weight*/*height^2^, a deterministic function of height and weight). This disciplined feature set was chosen because the unrestricted pool of all available IMU features produced ElasticNet over-fitting at *N* = 78, sacrificing roughly 0.10 *R*^2^ on percent body fat and roughly 0.08 on dominant grip strength relative to the curated version. Gender, age, ground-truth DXA and grip-strength outcomes, and identifier columns are excluded from the predictor pool. For percent body fat, the model additionally receives the model-derived feature ${\text{pbf}}_{\text{pred}}=100\cdot \left(0.98-\hat{\text{lean}}\text{/weight}\right)$, where $\hat{\text{lean}}$ is the per-fold leave-one-out prediction of lean mass; no ground-truth outcome is ever an input.
3. *Count-only*: the 30-second chair stand repetition count as the sole predictor, the conventional clinical score.
4. *Height + Weight*: height and weight only, the passive body-size information available without any test performance. This baseline isolates how much of each outcome is predictable from the two anthropometric inputs that also enter the mechanical and inverse-kinematics features used by the all-features model.

#### Model fitting and validation.

For all conditions, an ElasticNet regression was fitted with hyperparameters (regularization strength *α* and L1 ratio) selected by 3-fold inner cross-validation (ElasticNetCV). The demographic-free IMU-only condition is the single exception: because its movement features are numerous and strongly collinear with no dominant predictor, ElasticNet selection is unstable at *N* = 78 and overfits, so that condition uses ridge regression (RidgeCV), which jointly shrinks the correlated movement features and recovers their shared signal. Feature selection occurred inside each leave-one-out (LOO) fold, not before it, to prevent data leakage. Reported metrics are LOO *R*^2^, $R\left(=\sqrt{R^{2}}\right)$, and mean absolute error (MAE). All feature values were standardized (zero mean, unit variance) within each training fold using the held-out fold’s own scaler parameters.

#### Comparison of model conditions.

To test whether the IMU-only condition produced a significantly higher correlation than either the count-only or height-and-weight baseline, Steiger’s Z-test for dependent correlations [17] was applied using the Meng–Rosenthal–Rubin (1992) correction. The test uses the LOO prediction vectors from two conditions alongside the observed outcomes; a two-tailed *p* < 0.05 was used as the significance threshold.

#### Feature importance and stability selection.

To characterize which features were most reliably predictive, stability selection [18] was performed for the IMU-only condition across all five targets, and additionally for the all-features condition restricted to the percent body fat target. In each of 50 bootstrap resamples (75% subsampling without replacement), an ElasticNet was fitted and the selected features (non-zero coefficients) were recorded. Stability selection frequency is the proportion of resamples in which a feature was selected; features above the 0.5 threshold are considered reliably selected. Features were grouped into the six interpretable movement categories described above, and mean stability selection frequency was computed per category to identify which *types* of movement information were most predictive.

#### Forward stepwise model complexity analysis.

To identify the minimal sufficient feature set, forward stepwise selection was performed for each target using the IMU feature pool. At each step, the candidate feature producing the greatest incremental LOO *R*^2^ gain was added to the model. This process was continued up to *k* = 10 features, using plain linear regression (not ElasticNet) so that model size is exact and the *R*^2^ change at each step is attributable to a single named feature. Unlike the stability-selection ranking, forward step-wise selection naturally accounts for feature redundancy: a second highly correlated feature will produce minimal marginal gain and will be deprioritised in favour of complementary features from other movement categories.

## Figures and Tables

**Table 1:** Leave-one-out cross-validated Pearson *R* and coefficient of determination *R*^2^ with 95% bootstrap CI, plus MAE, across the four predictor conditions for the three outcomes.

Outcome	Condition	*R*	95% CI (*R*)	*R* ^2^	MAE	*n*
Lean mass (lbs)	All features	0.918	(0.89, 0.95)	0.842	7.41	73
	IMU-only	0.476	(0.30, 0.63)	0.210	16.34	73
	Count-only	0.100	(−0.15, 0.37)	−0.009	19.02	78
	Height + Weight	0.903	(0.87, 0.94)	0.815	8.19	78
Body fat (%)	All features	0.629	(0.49, 0.74)	0.394	4.35	73
	IMU-only	0.503	(0.33, 0.65)	0.249	4.84	73
	Count-only	−0.155	(−0.35, 0.07)	−0.040	5.75	78
	Height + Weight	0.342	(0.16, 0.65)	0.081	5.35	78
HGS dominant (lbs)	All features	0.853	(0.75, 0.91)	0.728	4.76	73
	IMU-only	0.519	(0.29, 0.69)	0.267	8.03	73
	Count-only	0.246	(0.00, 0.49)	0.051	8.94	78
	Height + Weight	0.773	(0.68, 0.85)	0.596	5.98	78

All-features = curated demographic-free IMU features + height + weight + BMI (ElasticNet); IMU-only = curated demographic-free IMU features (33 for percent body fat; 37 including angular-velocity channels for lean mass and dominant grip strength), fit with ridge regression; Height + Weight = anthropometrics alone; Count-only = 30-second repetition count.

**Table 2:** Steiger Z-test for dependent correlations: demographic-free IMU-only model vs. the count-only and Height + Weight baselines.

Outcome	vs. baseline	*r*_IMU_	*r*_base_	*Z*	*p*
Lean mass	Count-only	+0.476	+0.117	2.71	0.007 *
Lean mass	Height + Weight	+0.476	+0.899	−5.95	0.000
Body fat (%)	Count-only	+0.503	−0.174	4.50	0.000 *
Body fat (%)	Height + Weight	+0.503	+0.359	1.11	0.269
HGS dominant	Count-only	+0.519	+0.274	2.34	0.019 *
HGS dominant	Height + Weight	+0.519	+0.770	−2.75	0.006

* marks IMU-only significantly higher (*p* < 0.05 and *r*_IMU_ > *r*_baseline_).

**Table 3:** Forward stepwise selection on the curated demographic-free IMU features for Lean mass (lbs). Plain linear regression; LOO Pearson *R* reported.

Step	Feature	Category	*R*	Δ*R*
1	gyro_jerk_mean	Other	0.469	+0.469
2	velocity_trend	Fatigue	0.540	+0.071
3	cycle_peak_power_surrogate_mean_pkg	Power/Impulse	0.563	+0.024
4	cycle_peak_power_surrogate_cv	Power/Impulse	0.576	+0.013
5	sit_time_mean	Speed	0.598	+0.022
6	gyro_mean	Other	0.610	+0.012
7	gyro_z_rms	Other	0.613	+0.003
8	time_for_5	Speed	0.615	+0.002
9	ik_fixedh_knee_angle_range	Kinematics	0.614	−0.001
10	ik_fixedh_knee_angle_mean	Kinematics	0.646	+0.032

**Table 4:** Forward stepwise selection on the curated demographic-free IMU features for Body fat (%). Plain linear regression; LOO Pearson *R* reported.

Step	Feature	Category	*R*	Δ*R*
1	rfd_range_mag	Power/Impulse	0.472	+0.472
2	stand_asymmetry_mean	Cycle/Consistency	0.514	+0.043
3	cycle_peak_power_surrogate_cv	Power/Impulse	0.556	+0.042
4	top_15p_accel_mag	Power/Impulse	0.596	+0.040
5	ik_fixedh_hip_angle_range	Kinematics	0.602	+0.006
6	max_mag_vel	Speed	0.604	+0.002
7	fatigue_delta	Fatigue	0.605	+0.001
8	fatigue_ratio	Fatigue	0.618	+0.013
9	velocity_trend	Fatigue	0.620	+0.002
10	ik_fixedh_hip_angle_mean	Kinematics	0.620	−0.000

**Table 5:** Forward stepwise selection on the curated demographic-free IMU features for HGS dominant (lbs). Plain linear regression; LOO Pearson *R* reported.

Step	Feature	Category	*R*	Δ*R*
1	cycle_peak_power_surrogate_mean_pkg	Power/Impulse	0.604	+0.604
2	gyro_mean	Other	0.652	+0.047
3	consistency	Smoothness	0.655	+0.004
4	sit_speed	Speed	0.671	+0.016
5	rise_time_mean	Speed	0.674	+0.002
6	time_for_5	Speed	0.675	+0.001
7	ik_fixedh_knee_angle_mean	Kinematics	0.674	−0.001
8	ik_fixedh_knee_angle_range	Kinematics	0.671	−0.003
9	rfd_range_mag	Power/Impulse	0.668	−0.003
10	rfd_std_mag	Power/Impulse	0.677	+0.009

**Table 6:** Participant characteristics (*N* = 78). Continuous variables reported as mean ± SD with range. Age is reported for participant characterisation only and was excluded from all predictive models.

Variable	Value (Mean ± STD)	Range
*N*	78	
age	51.49 ± 16.40	19.00–81.00
height	66.60 ± 5.02	59.30–88.40
weight	152.40 ± 33.77	93.90–284.20
bmi	24.08 ± 4.30	15.99–40.08
lean_mass	98.36 ± 24.56	55.96–167.23
LBMI totalJean/height^2^ (kg/m^2^)	15.42 ± 2.47	10.04–21.58
Body Fat (%)	32.81 ± 7.02	17.60–49.40
HGS Dominant (lbs)	35.91 ± 11.85	15.00–65.30
HGS Non-dominant (lbs)	33.42 ± 11.90	14.60–65.30
30s Chair Stands (reps)	16.94 ± 5.46	9.00–30.00
Gender (Female)	57	
Gender (Male)	21	

## Data Availability

The individual-level participant data generated and analysed during this study are not publicly available because the IRB-approved protocol does not include consent for public data release. De-identified summary data and a code-only reproducibility package are available from the corresponding author on reasonable request and with permission from the UCSD Institutional Review Board.

## Code Availability

The custom analysis code that supports the findings of this study (the feature-extraction, leave-one-out cross-validation, and figure-generation scripts) is available from the corresponding author on reasonable request.
